# Physical activity and socio-economic status of single and married urban adults: a cross-sectional study

**DOI:** 10.7717/peerj.12466

**Published:** 2021-11-09

**Authors:** Daniel Puciato, Michał Rozpara

**Affiliations:** 1 Faculty of Physical Education and Physiotherapy, Opole University of Technology, Opole, Opole, Poland; 2 Institute of Sport Sciences, The Jerzy Kukuczka Academy of Physical Education, Katowice, Silesia, Poland

**Keywords:** Physical activity, Socio-economic status, Marital status, Metropolitan population

## Abstract

**Background:**

Changing family models have resulted in a large increase in the number of single-person households. This phenomenon has certain implications for society and the economy as single people often exhibit different behaviours, including their engagement in health-related physical activity, than those who are married and living with partners. However, the results of studies on determinants of physical activity in people of different marital status have been inconclusive. The aim of this study was to identify associations between physical activity and socioeconomic status in single and married urban adults.

**Methods:**

The study material consisted of 4,460 persons (1,828 single and 2,632 married and living with partners). A cross-sectional study was conducted in Wroclaw (Poland). A diagnostic survey-direct interview method was used. Two research tools were applied: the International Physical Activity Questionnaire Short Form (IPAQ-SF) and the Socioeconomic Status Questionnaire (S-ESQ). The level of respondents’ physical activity was assessed following WHO recommendations. The descriptive statistics included the number and frequency of categories of dependent and independent variables as well as measures of association between them, *i.e*., crude and adjusted odds ratios.

**Results:**

The odds ratio of meeting the WHO physical activity recommendations was almost 70% higher in single than in married respondents (OR = 1.67; CI [1.46–2.19]), and slightly more than 40% higher after adjusting for sex, age and education (aOR = 1.42; CI [1.21–1.67]). In both groups socioeconomic the respondents’ status revealed a significant and slightly different association with their levels of physical activity. Occupational status and financial savings significantly affected the level of physical activity in single respondents, while net disposable income was a significant modifier of physical activity levels in respondents who were married or lived with their partners.

**Conclusions:**

Assessment of the levels and determinants of physical activity among people of different marital status should be continued and extended to other subpopulations. This will allow effective preventive and therapeutic measures to be taken for groups most at risk of hypokinesia. Programs aimed at improving physical activity accounting for the socioeconomic status and marital status of their beneficiaries are particularly important.

## Introduction

Changing family patterns have resulted in a large increase in the number of single-person households. Between 1980 and 2015, the number of single-person households worldwide increased two and a half times: from about 118 million to 300 million. Projections show that single-person households will be the fastest growing household type by 2030, with their number expected to increase by another 120 million. The share of single-person households in Poland is growing and amounts to about 24% (Eurostat, 2019). This demographic phenomenon is driven by the younger generation, and the increasing number of divorced and widowed elderly people is particularly evident in metropolitan environments (Dąbrowska et al., 2018; Villanueva, Rubies & Perez, 2019).

Despite the increasing number of single adults, especially in developed countries, very little is known about their health behaviours in terms of habitual physical activity, diet, stress avoidance and management, and reduction of stimulant use compared to those who are married or live with their partners. Yet, lifestyle remains the most important determinant of health status, and its key component is physical activity, which can be defined as any bodily movement produced by skeletal muscles that results in energy expenditure above the resting metabolic rate (Caspersen, Powell & Christenson, 1985). Total physical activity, which is the subject of this study, is the sum of leisure-time, domestic, occupational and transportation-related physical activity. While, according to “the physical activity paradox” of Holtermann et al. (2009), high levels of physical exercise, especially static, during occupational activities, do not always have a positive impact on health, appropriately selected physical activities in other areas of life, mainly in leisure time, are of considerable importance in the prevention and treatment of diseases that are frequently the cause of premature death (Mynarski et al., 2012; Williamson et al., 2016). The positive relationships between leisure-time physical activity and physical fitness and performance have also been well documented (Rożek-Piechura et al., 2014). Studies have confirmed the positive effects of leisure-time physical exercise on mood, cognitive processes, optimism and quality of life (Dziubek et al., 2016; Pavey, Burton & Brown, 2015). However, physical exercise of appropriate frequency, duration, and intensity, can not only serve preventive but also rehabilitative functions, allowing the return to full working capacity and activity after illness, injury, or fatigue (Arokoski, Juntunen & Luikku, 2002).

The results of previous studies on physical activity among people of different marital status are inconclusive. Some studies have indicated higher total physical activity levels among single adults compared to those in relationships (Bassett et al., 2010; Del Duca et al., 2013; Garcez et al., 2015; Gul et al., 2019; Malambo et al., 2016; Rapp & Schneider, 2013). Similar observations regarding physical activity associated with active transport were made by Zeinab (2019). Schneider & Becker (2005) showed that single and widowed persons were the most, and divorced and married persons were the least physically active.

However, authors also noted higher levels of physical activity among individuals in relationships (Pardo et al., 2014; Park et al., 2019). Lakka, Kauhanen & Salonen (1996) found higher physical activity levels in married men compared to single men. The observed differences, however, were not attributed to the duration of physical activity but rather to its intensity. Men in relationships performed shorter but more intensive physical activity, whereas single men preferred physical activity of slightly longer duration but lower intensity. In some studies, the direction of correlation between physical activity and marital status depended on respondents’ sex. Drygas et al. (2013) reported that as far as women were concerned the highest physical activity levels were found in unmarried women, followed by divorcees and married women, while the lowest were noted in widows. On the other hand, divorced men were found to be the most physically active, followed by married and single men, while widowers were the least physically active. Sex also determined the direction of the relationship of physical activity with marital status in Kwaśniewska et al. (2016) who reported the highest level of physical activity among widows, followed by divorced and married women, while unmarried women were characterized by the lowest level of physical activity. Among men, single male adults were the most active, followed by married men and widowers, while divorced men were the least active. In contrast, Wang et al. (2011) found significant associations between physical activity and marital status only in women. In their study, married women were more physically active than divorcees and widows. Dlugonski & Motl (2013) and Peralta et al. (2017) arrived at similar conclusions. The results of their studies showed that married women with children were more physically active than both married women without children and unmarried women. Some previous studies also revealed statistically non-significant differences in physical activity levels between single and married persons (Biernat, 2015; Lusmägi, Einasto & Roosmaa, 2016; Macek et al., 2019*; Marques et al., 2016)*.

The reviewed studies therefore indicate certain inconsistencies in regard to exactly how sex determines the direction of the relationship between physical activity and marital status. The differences in study results are probably due to both the analysis of different domains of physical activity and the use of different measurement methods and tools. It can be hypothesized that cultural factors may also be of significance here. However, the verification of this hypothesis requires further research in an international context. The aforementioned literature review reveals that it is still unclear whether the level of total physical activity in single individuals and those in relationships differs significantly.

It is also unknown whether physical activity levels are affected by socioeconomic factors other than marital status, such as occupation or financial status. The results of previous studies on physical activity levels in different occupational groups were inconclusive. Wu & Porell (2000) observed higher levels of leisure-time physical activity in white-collar workers compared to blue-collar workers. Chen et al. (2015) and Puciato et al. (2013), on the other hand, found the highest levels of total physical activity in blue-collar workers, and the lowest in white-collar workers and the unemployed. Schneider & Becker (2005) in their study of working-age individuals from Germany found that the self-employed reported the highest leisure-time physical activity levels, while white-collar and blue-collar workers the lowest. In contrast, high levels of physical activity among college students were found by Bergier et al. (2018), Mynarski et al. (2009) and Nawrocka et al. (2012). The potential relationships of physical activity with financial situation are also ambiguous. Positive correlations of total physical activity with the financial situation of people of working age were reported by Biernat (2015), Jurakic et al. (2014) and Puciato (2016). Kaewthummanukul & Brown (2006) and Van Stralen et al. (2009) observed that income situation is not significantly associated with leisure-time physical activity, while Sallis et al. (2016) noted negative associations between income level and total physical activity level of adults.

Furthermore, Beenackers et al. (2012) and Stalsberg & Pedersen (2018) also suggest that when considering potential associations of physical activity and socioeconomic status, it is important to distinguish the location of physical exercise. Indeed, while the associations of leisure-time physical activity with socioeconomic factors were evident in previous studies, they were inconclusive with respect to total physical activity, which is likely due in part to the complexity of this particular variable.

Previous research on the relationships between physical activity and socioeconomic status has not been conducted separately for single and married adults, and this represents a significant research gap. Consequently, an interesting and open research problem is whether socioeconomic modifiers of physical activity are similar in single adults and those in relationships. Addressing this research question is warranted as findings from previous studies suggest significant differences between both groups on issues such as consumer behaviours (Dąbrowska et al., 2018), healthcare expenditure (Tur-Sinai, Magnezi & Grinvald-Fogel, 2018) or food-related lifestyles (Kim, Lee & Lee, 2018). Young & Lachapelle (2017) also noted some differences in transportation behaviours between the two groups, which is relevant to the subject of the present study. Single adults often pursue a more environmentally sustainable lifestyle with respect to travel. On average, they commute shorter distances to work and use a private car less often than respondents in relationships. There are also some differences between single and married individuals with respect to their financial situation. Single-person households do not have multiple sources of income, nor do they have the same access to welfare programs as, for example, families with children. The size and structure of expenditures of single adults is also different, as they do not usually have to make financial arrangements for other family members, including children. Surveys of single-person households in the U.S. also suggest that single people work longer and save more than married people of similar socioeconomic status (Lim, 2019). Thus, on the one hand, they have less free time to devote to physical activity, while on the other hand, they work longer on average, which translates into higher levels of their occupational physical activity. In addition, higher average *per capita* income and savings mean that single adults are more financially able than those in relationships to afford to pay for physical recreation, purchase sports equipment or nutritional supplements.

The aim of the present study was to identify the relationships between the level of total physical activity and socio-economic status in single and married urban adults. The following research questions were addressed in the study:

1. What is the level of physical activity self-reported by single and married adults?
2. Do occupational status, steady income, *per capita* income, net disposable income, savings and indebtedness affect respondents’ physical activity levels?
3. Is the impact of socioeconomic modifiers of physical activity similar in single persons and persons in relationships?

## Materials & methods

### Participants

The study was conducted between 2014–2016 in Wrocław, the fourth largest city of Poland, with a population of about 632,000. The sample selection for the study was conducted as described earlier in Puciato & Rozpara (2020). The following criteria were adopted for the inclusion of participants in the study: address of residence; working age; and lack of chronic diseases such as cancer, osteoporosis, arthritis, diabetes, hypertension. The exclusion criteria were residence other than one of the selected streets, pre- or post-working age, and the presence of chronic disease. All respondents were informed about the purpose, conduct of the study and voluntary participation in the study. All respondents gave their informed and voluntary verbal consent to participate in the study. The study design received a positive evaluation from the Commission of Bioethics of the University School of Physical Education in Wrocaw. The mean refusal rate was approximately 4.6%. The minimum sample size was estimated using the formula (Brzeziński, 2011):

$${n}= \displaystyle{{ N} \over {{1 + }\displaystyle{{{{ e}^{\rm 2}}{ (N - 1)}} \over {{ z}_{ \alpha }^{\rm 2}{ pq}}}}}$$

where: *N—*number of Wrocław residents on December 31, 2013 (*N* = 632 067); *p—*fraction of working-age Wrocław residents on December 31, 2013 (*p* = 0.63); *q—*constant calculated as 1 − *p* (*q* = 0.37), *e—*standard error of estimate of the fraction (*e* = 1.5), *u*_α_—value of standardized normal distribution *N*(0, 1) for confidence interval of 1 − α (z_α_ = 1.96 for α = 0.05). The study material consisted of 4,460 respondents, including 2,331 women and 2,129 men. A total of 1,828 respondents were single (1,000 women, 828 men) and 2,632 were married or in relationship with their partners (1,331 women, 1,301 men).

The division of respondents into two groups was based on the *de facto* marital status as defined in European public statistics (Central Statistical Office, 2011). Group I (single persons) included single, widowed, divorced, separated and legally separated persons as well as persons not living in a consensual union with another person, legally married persons but not forming a *de facto* marriage and not living in a consensual union with another person. Group II included married persons and persons living with their partners. The respondents’ characteristics are presented in Table 1.

**Table 1:** Socio-demographic characteristics of Wrocław respondents.

Variable	Total (*n* = 4460)				Women (*n* = 2331)				Men (*n* = 2129)			
	Group I (*n* = 1,828)		Group II (*n* = 2,632)		Group I (*n* = 1,000)		Group II (*n* = 1,331)		Group I (*n* = 828)		Group II (*n* = 1,301)	
	f	p	f	p	f	p	f	p	f	p	f	p
Age												
18–24 years	508	27.8	112	4.3	257	25.7	60	4.5	251	30.3	52	4.0
25–34 years	818	44.7	371	14.1	423	42.3	186	14.0	395	47.7	185	14.2
35–44 years	190	10.4	692	26.3	75	7.5	369	27.7	115	13.9	323	24.8
45–54 years	133	7.3	623	23.7	100	10.0	296	22.2	33	4.0	327	25.1
55–64 years	179	9.8	834	31.7	145	14.5	420	31.6	34	4.1	414	31.8
Education												
Primary	651	35.6	1,089	41.4	323	32.3	479	36.0	328	39.6	610	46.9
Secondary	834	45.6	783	29.7	507	50.7	489	36.7	327	39.5	294	22.6
Higher	343	18.8	760	28.9	170	17.0	363	27.3	173	20.9	397	30.5
Job												
Unemployed	253	13.8	433	16.5	157	15.7	329	24.7	96	11.6	104	8.0
Labourer	488	26.7	677	25.7	183	18.3	241	18.1	305	36.8	436	33.5
White collar worker	452	24.7	904	34.3	274	27.4	490	36.8	178	21.5	414	31.8
Businessman	107	5.9	509	19.3	31	3.1	185	13.9	76	9.2	324	24.9
Student	528	28.9	109	4.1	355	35.5	86	6.5	173	20.9	23	1.8
Steady income												
Yes	1,318	72.1	2,290	87.0	689	68.9	1,122	84.3	629	76.0	1,168	89.8
No	510	27.9	342	13.0	311	31.1	209	15.7	199	24.0	133	10.2
*Per capita* income												
<260 USD	545	29.8	670	25.5	336	33.6	415	31.2	209	25.2	255	19.6
≥260 USD	1,283	70.2	1,962	74.5	664	66.4	916	68.8	619	74.8	1,046	80.4
Net income												
No income	213	11.7	178	6.8	121	12.1	79	5.9	92	11.1	99	7.6
<104 USD	852	46.6	1,150	43.7	525	52.5	688	51.7	327	39.5	462	35.5
≥104 USD	763	41.7	1,304	49.5	354	35.4	564	42.4	409	49.4	740	56.9
Savings												
No	1,030	56.3	1,302	49.5	609	60.9	765	57.5	421	50.8	537	41.3
Yes	798	43.7	1,330	50.5	391	39.1	566	42.5	407	49.2	764	58.7
Indebtedness												
Yes	735	40.2	1,372	52.1	413	41.3	636	47.8	322	38.9	736	56.6
No	1,093	59.8	1,260	47.9	587	58.7	695	52.2	506	61.1	565	43.4

**Notes**:

Group I: Single, widowed, divorced, separated and legally separated persons as well as persons not living in a consensual union with another person, legally married persons but not forming a *de facto* marriage and not living in a consensual union with another person; Group II: Married persons and persons living with partners.

f, frequency; p, percentage.

### Assessment of physical activity and socioeconomic status

The study was cross-sectional. The diagnostic survey-direct interview method was applied. Two research tools were used: International Physical Activity Questionnaire (International Physical Activity Questionnaire (IPAQ), 2014) and Socioeconomic Status Questionnaire (S-ESQ). The diagnostic survey was used to measure the habitual physical activity of the Wrocław residents. The International Physical Activity Questionnaire—Short Form (IPAQ-SF) was used to provide information about the frequency (day/week) and time (min/day) of physical activity at three intensity levels (vigorous, moderate, light) in four domains together: leisure time, occupational activities, transportation-related activities and domestic activities. Based on data on the duration of physical activity of vigorous and moderate intensity and World Health Organization (WHO) (2010), the level of physical activity was assessed as a dependent variable (DV). Two groups of respondents were distinguished:

1. Respondents meeting WHO recommendations (sufficiently physically active for health enhancement)—who, during the week, were physically active for at least 150 min (moderate-intensity exercises) or for at least 75 min (vigorous-intensity exercises), if no single exercise lasted less than 10 min.
2. Respondents not meeting WHO recommendations (insufficiently physically active for health enhancement)—who engaged in physical activity of less than 150 min (moderate intensity) or 75 min (vigorous intensity) per week.

The other research tool was the socio-economic status questionnaire for people of working age (S-ESQ) developed by one of the authors of this paper, based on literature analysis and his own professional experience. Data on the physical activity of the Wrocław residents were supported with information on their marital status (Group I, Group II), occupational status (unemployed, labourer, white collar worker, businessman, student), steady income (yes, no), *per capita* income (up to 260 USD, over 260 USD), net disposable income (not income up to 104 USD, over 104 USD), having money savings (no, yes), and having debt (no, yes) as independent variables (IVs); and sex (female, male), age (18–24 years, 25–34 years, 35–44 years, 45–54 years, 55–64 years), education (primary, secondary, higher) as confounding variables (CVs). The basis for assigning the respondents to particular occupational groups was the Regulation of the Polish Minister of Labour and Social Policy of 27 April 2010 on the Classification of Professions and Specialities for the Labour Market Needs and the Scope of its Application. Journal of Laws of 2010, No. 82, item 537 (*Regulation…, 2010*). The questionnaire was pilot tested in accordance with the methodology described in literature (Babbie, 2008), and based on the feedback from respondents all perceived errors and ambiguities were corrected in the questionnaire.

### Statistical analysis

For the variables under consideration, the frequency (f) and percentage (p) of respondents in particular categories were determined. The assessment of relationships between the level of physical activity and socio-economic status was based on crude odds ratio (OR) and adjusted odds ratio (aOR). The study used a modeling variant that assumed independent effects of IVs on DV without assessing the interaction factor between IVs, which were not measured due to the small sample size. The OR for specific IVs was adjusted for the effect of CVs. The predictive value of the analyzed models was assessed with the area under the ROC curve (AUC). All analyses were performed separately for single respondents and respondents in relationships. This research approach was considered valid given the purpose of the study and its cross-sectional character (Tamhane et al., 2016). The level of statistical significance was set at α = 0.05. All statistical calculations were made using the IBM SPSS Statistics 26 software package (IBM Corporation, Armonk, NY, USA).

## Results

Table 2 presents the results of the study demonstrating the level of physical activity of single and married Wrocław residents. Among the single Wrocław residents, 75% met and 25% did not meet the WHO physical activity recommendations. In the group of married respondents, 64% were sufficiently and 36% were not sufficiently physically active. The odds of meeting the WHO recommendations were nearly 70% higher in single respondents (OR = 1.67; CI [1.46–2.19]) than in married respondents, and slightly more than 40% higher (aOR = 1.42; CI [1.21–1.67]) after adjusting for sex, age, and education (Table 2). Tables 3 and 4 present the analysis of relationships between respondents’ levels of physical activity and socio-economic factors. The odds ratio (OR) and confidence interval (CI) were given as crude values (Model 1) and adjusted values: sex (Model 2); sex and age (Model 3); and sex, age and education (Model 4). Based on the AUC statistic successive models had increasingly higher predictive values (index of discrimination), *i.e*., their ability to correctly classify occurrences of the condition under consideration (individuals meeting the WHO physical activity recommendations) and the absence of its occurrences (individuals not meeting the WHO physical activity recommendations) for individual independent variables increased. However, it should be noted that none of the AUC predictive value indices exceeded the magnitude of 0.7, which indicates that models based on a single independent variable have a low capability of classifying subjects into groups that meet and do not meet the WHO physical activity recommendations.

**Table 2:** Physical activity of single and married respondents from Wrocław in view of WHO physical activity 2 recommendations.

Marital status	Meeting recommendations		Not meeting recommendations		Model 1 OR [95% CI]	Model 2 aOR [95%CI]^a^	Model 3 aOR [95% CI]^b^	Model 4 aOR [95% CI]^c^
	f	p	f	p				
Group I	1,372	75.1	456	24.9	1.67*** [1.46–1.90]	1.68*** [1.48–1.92]	1.44*** [1.23–1.69]	1.42*** [1.21–1.67]
Group II	1,694	64.4	938	35.6	1.00	1.00	1.00	1.00

**Notes:**

Group I: Single, widowed, divorced, separated and legally separated persons as well as persons not living in a consensual union with another person, legally married persons but not forming a *de facto* marriage and not living in a consensual union with another person; Group II: Married persons and living partners.

OR, Crude odds ratio; aOR, Adjusted odds ratio (the Cochran–Mantel–Haenszel test); CI, Confidence interval for OR.

a sex.

b sex, age.

c sex, age, education.

*** *p* ≤ 0.001.

**Table 3:** Physical activity and socio-economic status of single Wrocław residents.

Variable	Model 1 OR [95% CI]	Model 2 aOR [95% CI]^a^	Model 3 aOR [95% CI]^b^	Model 4 aOR [95% CI]^c^
Job	AUC = 0.573	AUC = 0.593	AUC = 0.607	AUC = 0.624
Unemployed	1.08 [0.73–1.58]	1.07 [0.72–1.57]	0.71 [0.47–1.08]	0.84 [0.52–1.36]
Labourer	0.60*** [0.44–0.80]	0.53*** [0.39–0.72]	0.59** [0.42–0.84]	0.87 [0.56–1.34]
White collar worker	0.56*** [0.41–0.75]	0.53*** [0.40–0.72]	0.57** [0.39–0.84]	0.61* [0.39–0.96]
Businessman	0.56* [0.35–0.90]	0.44** [0.27–0.73]	0.40** [0.22–0.72]	0.41* [0.20–0.86]
Student	1.00	1.00	1.00	1.00
Steady income	AUC = 0.535	AUC = 0.554	AUC = 0.595	AUC = 0.622
Yes	0.69** [0.54–0.89]	0.68** [0.53–0.87]	0.77 [0.59–1.01]	0.78 [0.60–1.02]
No	1.00	1.00	1.00	1.00
*Per capita* income	AUC = 0.517	AUC = 0.537	AUC = 0.589	AUC = 0.618
<260 USD	1.19 [0.94–1.50]	1.21 [0.96–1.54]	1.05 [0.82–1.34]	0.98 [0.76–1.26]
≥260 USD	1.00	1.00	1.00	1.00
Net income	AUC = 0.518	AUC = 0.541	AUC = 0.596	AUC = 0.623
No income	1.42 [0.97–2.06]	1.47* [1.01–2.14]	1.43 [0.96–2.12]	1.43 [0.97–2.10]
<104 USD	1.02 [0.81–1.27]	1.05 [0.84–1.32]	1.03 [0.82–1.30]	1.01 [0.79–1.29]
≥104 USD	1.00	1.00	1.00	1.00
Savings	AUC = 0.532	AUC = 0.544	AUC = 0.597	AUC = 0.619
No	0.77* [0.62–0.95]	0.78* [0.63–0.97]	0.77* [0.62–0.96]	0.69** [0.54–0.86]
Yes	1.00	1.00	1.00	1.00
Indebtedness	AUC = 0.529	AUC = 0.547	AUC = 0.594	AUC = 0.617
Yes	0.79* [0.64–0.98]	0.80* [0.64–0.98]	0.81 [0.65–1.02]	0.90 [0.72–1.13]
No	1.00	1.00	1.00	1.00

**Notes:**

OR, Crude odds ratio; aOR Adjusted odds ratio (Mantel–Haenszel odds ratio); CI, Confidence interval for OR; AUC, Area under the ROC curve.

a sex.

b sex, age.

c sex, age, education.

* *p* ≤ 0.05.

** *p* ≤ 0.01.

*** *p* ≤ 0.001.

**Table 4:** Physical activity and socio-economic status of married Wrocław residents.

Variable	Model 1 OR [95% CI]	Model 2 aOR [95% CI]^a^	Model 3 aOR [95% CI]^b^	Model 4 aOR [95% CI]^c^
Job	AUC = 0.536	AUC = 0.536	AUC = 0.553	AUC = 0.606
Unemployed	0.63* [0.40–0.98]	0.58* [0.37–0.92]	0.96 [0.49–1.86]	1.05 [0.49–2.27]
Labourer	0.98 [0.63–1.50]	1.01 [0.64–1.60]	1.23 [0.61–2.49]	1.32 [0.51–3.40]
White collar worker	0.88 [0.58–1.35]	0.77 [0.50–1.18]	0.76 [0.42–1.37]	1.02 [0.52–1.99]
Businessman	0.86 [0.56–1.34]	1.13 [0.71–1.81]	0.57 [0.23–1.39]	0.75 [0.29–1.91]
Student	1.00	1.00	1.00	1.00
Steady income	AUC = 0.511	AUC = 0.536	AUC = 0.559	AUC = 0.597
Yes	1.21 [0.96–1.52]	1.17 [0.93–1.49]	1.19 [0.93–1.51]	0.91 [0.70–1.18]
No	1.00	1.00	1.00	1.00
*Per capita* income	AUC = 0.504	AUC = 0.535	AUC = 0.564	AUC = 0.599
<260 USD	1.04 [0.87–1.25]	1.09 [0.90–1.31]	1.08 [0.90–1.3]	1.13 [0.93–1.38]
≥260 USD	1.00	1.00	1.00	1.00
Net income	AUC = 0.579	AUC = 0.587	AUC = 0.610	AUC = 0.626
No income	1.94** [1.31–2.86]	1.94** [1.31–2.88]	2.08*** [1.39–3.10]	1.55* [1.01–2.39]
<104 USD	0.60*** [0.51–0.71]	0.62*** [0.52–0.73]	0.58*** [0.49–0.69]	0.61*** [0.50–0.73]
≥104 USD	1.00	1.00	1.00	1.00
Savings	AUC = 0.506	AUC = 0.531	AUC = 0.557	AUC = 0.598
No	0.95 [0.81–1.12]	0.99 [0.85–1.17]	1.02 [0.87–1.20]	0.99 [0.83–1.18]
Yes	1.00	1.00	1.00	1.00
Indebtedness	AUC = 0.508	AUC = 0.534	AUC = 0.561	AUC = 0.596
Yes	1.07 [0.91–1.25]	1.05 [0.89–1.23]	1.07 [0.91–1.26]	1.11 [0.94–1.31]
No	1.00	1.00	1.00	1.00

**Notes:**

OR, Crude odds ratio; aOR Adjusted odds ratio (Mantel–Haenszel odds ratio); CI, Confidence interval for OR; AUC, Area under the ROC curve.

a sex.

b sex, age.

c sex, age, education.

* *p* ≤ 0.05.

** *p* ≤ 0.01.

*** *p* ≤ 0.001.

Among the single respondents there were significant associations between physical activity levels and occupational status, steady income, savings and debt assessed independently of sex, age and education (Table 3). The adjusted odds ratio of meeting the WHO recommendations for single businessmen was almost 60% (aOR = 0.41; CI [0.20–0.86]), and for white-collar workers almost 40% (aOR = 0.61; CI [0.39–0.96]) lower than that of college students when their sex, age and education were considered. Single respondents without money savings were more than 30% (aOR = 0.69; CI [0.54–0.86]) less likely to reach a WHO-recommended physical activity level compared to those of the same sex, age, and education with savings (Table 3).

Also, models demonstrating associations of physical activity level with socioeconomic factors in married respondents showed poor predictive values (Table 4). In married respondents physical activity levels were statistically significantly associated with occupation and net disposable income regardless of sex, age or education. The adjusted odds ratio of achieving sufficient physical activity levels following WHO recommendations was 55% higher (aOR = 1.55; CI [1.01–2.39]) for those with no disposable income and 39% lower (aOR = 0.61; CI [0.50–0.73]) for those with an income of less than $104 than for respondents of the same sex, age and education with a monthly net disposable income of at least $104 (Table 4).

## Discussion

Among the single respondents, the conditional probability of fulfilling the WHO overall health-enhancing physical activity recommendations was higher than among the married respondents (Table 2). Similar study results were also reported by Del Duca et al. (2013), Gul et al. (2019) and Malambo et al. (2016). On the other hand, Rapp & Schneider (*2013*), after adjusting their study results for age, measurement period, and having children, observed negative effects on weekly physical activity levels for men and women. The effects were strongest for legal marriages, while slightly weaker for consensual unions. One of the potential reasons for these findings among the respondents may be that single persons do not have to perform tasks related to family life, including childcare. Consequently, they often have more free time at their disposal than people in relationships of a similar socio-economic status. They may also devote part of this surplus leisure time to the pursuit of physical activities. This assumption was supported by Bassett et al. (2010) and Garcez et al. (2015), who revealed that leisure time physical activity levels in people living alone were higher than in people in relationships. In addition, Zeinab (2019) reported higher levels of active transportation in single people than in people in relationships. Using bicycles, public transportation, or walking requires more time than driving, so again single adults are mostly at an advantage. This is confirmed by Young & Lachapelle (2017), who found that single persons are less likely than married persons to use private cars. This is also facilitated by the metropolitan environment, where infrastructure and public transport facilities are better developed than outside cities. In addition, as Lim (2019) demonstrated, single persons tend to work more than those in relationships, which would also indicate higher work activity levels among the former. However, this hypothesis should be approached with caution as some studies indicate that higher levels of occupational physical activity are, in fact, found among married people (Jurakic et al., 2014; Puciato et al., 2013). This is because they often have to work more than their single counterparts in order to financially support not only themselves but also other family members, for example, children. This is particularly evident in countries with a medium level of economic development, such as Poland, where wages and purchasing power are lower than in the most economically developed countries. It is also important to remember that high levels of occupational physical activity do not always have a positive impact on health and quality of life (Holtermann et al., 2009). Considering the results of the present study and those by other authors it can be assumed that higher levels of leisure and transport-related physical activity in singles are mainly associated with higher odds of meeting WHO physical activity standards in single than in married persons. However, this hypothesis requires further research with the use of tools enabling the measurement of physical activity in specific domains.

Significant correlations of physical activity with some socioeconomic status variables were found among the respondents. Associations of physical activity with occupational status and having savings were reported in single respondents (Table 3), and in married respondents with net disposable income (Table 4).

Among the single respondents from Wrocław, college students were the most likely while the self-employed and white-collar workers were the least likely to meet WHO physical activity recommendations (Table 3). The relatively high physical activity among the students is not surprising and was also previously observed by Bergier et al. (2018) and Mynarski et al. (2009). On the other hand, Wilson et al. (2020) reported high levels of transportation-related physical activity in college students. Students are mostly characterized by good health and ample leisure time resources which makes them quite free to pursue various forms of physical activity. Moreover, students’ frequent participation in physical activities within the framework of institutionalized physical education, tourist associations or academic sports associations, provides them with good conditions for performing physical exercises. The relatively low odds of white-collar workers meeting the physical activity recommendations in the present study were also confirmed in previous studies (Nawrocka et al., 2012; Nawrocka et al., 2017; Puciato et al., 2013). The nature of the tasks performed by representatives of these occupations means that the amount of physical exercise carried out during their professional duties and commuting is mostly not very high. Also, the duration of working time, primarily among businessmen, significantly limits the possibility to pursue leisure-time and domestic physical activities (Gu et al., 2016; Jurakic et al., 2014). In addition, Vandelanotte et al. (2015) found that the higher leisure-time physical activity of white-collar workers compared to blue-collar workers did not compensate for the former’s occupational physical activity. They also found that occupational physical activity was the most important component of total physical activity, which was lower in white-collar workers than in blue-collar workers. This effect is even more pronounced in single people, who, according to Lim (2019), work longer hours than married people in a similar socio-economic situation. The same study also revealed that single people attach more importance to having savings than married people. Not surprisingly, among the single respondents, the odds of meeting the WHO recommendation of health-enhancing physical activity were higher among those with money savings (Table 3). Although previous studies did not address the relationship between physical activity and savings, it can be assumed that individuals with savings are more affluent than those without. On the other hand, positive associations of physical activity with the financial situation of people of working age were observed in previous studies (Biernat, 2015; Jurakic et al., 2014; Puciato, 2016). A potential reason for the high levels of physical activity among single adults with savings may also be that frugal individuals possess certain character traits, that is: thriftiness, a sense of control over their lives, or foresightedness. Some prior research suggests that these traits may in some way determine health behaviours, including physical activity (McAuley & Blissmer, 2000). Having savings may also be associated with a sense of security, better social functioning, and better quality of life, which is also not insignificant for physical activity levels (Pucci et al., 2012; Zeinab, 2019).

Among the married respondents, the odds of meeting the WHO physical activity recommendation were the highest for those without net disposable income and the lowest for respondents with small net disposable income (Table 4). The observed directions of these associations may be both a peculiar artefact as well as a result of certain objective premises. The need for extra professional duties, independent tasks related to household management and childcare, or the use of cheaper forms of transportation (walking, cycling, public transportation) by people in a poor financial situation may explain their relatively high levels of occupational and transportation-related physical activity. Moreover, as shown by Borodulin et al. (2016), expenditures related to the pursuit of leisure-time exercise-related physical activity are a significant barrier only for the unemployed, of whom there were only a dozen or so percent among the respondents. Consequently, the lack of net disposable income among some of the respondents did not necessarily make a significant difference in the level of their leisure-time physical activity. Net disposable income itself has not previously been considered a potential determinant of physical activity, which greatly constrains the discussion. However, as previously mentioned, a large body of previous research has indicated a positive effect of the amount of total income on physical activity levels (Biernat, 2015; Jurakic et al., 2014; Puciato, 2016). Therefore, the question of relationship between physical activity and net disposable income should still be considered open.

Another important observation is the differences in the correlations between total physical activity and socio-economic factors in single and married adults. The correlations were more evident in the former. They concerned both occupational and financial status. Among the married respondents, significant correlations were found only between physical activity and net disposable income. Moreover, the highest odds of meeting the WHO physical activity recommendations were noted among married respondents with no net disposable income. It should also be emphasized that although the OR values of many of the models considered were statistically significant, these models were characterized by rather low predictive values. It should be explained by the fact that the study analysed models of associations between PA and specific indices of socio-economic status (simple regression models) without considering interactions between them or other factors of the level of physical activity of the studied Wrocław residents, for example, their physical fitness. Consequently, this still open research problem requires further research.

### Limitations

The study has its strengths and weaknesses. The strengths of the paper include the division of the respondents by marital status, since the assessment of relationships between physical activity and socioeconomic status has not previously been conducted separately for single and married individuals. Also, economic factors such as net disposable income, savings or debt as potential predictors of physical activity have not been considered before. The study thus represents an important contribution to the existing state of science, particularly since it reports significant correlations between quality of life with net disposable income and money savings. The weaknesses of the study include its cross-sectional character and the spatial limitation to the inhabitants of one city only. Consequently, the study results cannot be applied to the general population, even more so since the predictive values of the obtained models was low. In future research, the spatial scope should be extended to whole territory of Poland and other Central and Eastern European countries. Further studies should also be continuous, for example, cohort surveys, and take into account the effects of changes in marital status on the level and structure of physical activity. In addition, it is worthwhile to use research tools that measure physical activity in specific domains, that is: leisure time, work, at home and while moving. The strength and direction of correlations between physical activity and socio-economic factors can vary. Sub-categories of single respondents, that is, single women and men, widows and widowers, divorced people, married people and living with partners, should also be included in future studies. As authors of some previous studies have shown, the impact of marital status on physical activity levels can vary.

## Conclusions

Single respondents were more likely to meet the WHO recommendations for total physical activity than married respondents. The study also revealed significant relationships of physical activity with occupational status and savings (in single persons) and with net disposable income (in married persons).

The results of the present study warrant the conclusion that the measurement of the levels and determinants of physical activity in people of different marital status should be continued and extended to other sub-populations. This will allow effective prophylactic and therapeutic measures to be taken for groups most at risk of hypokinesia. Programs aimed at improving physical activity seem to be particularly important and should be directed to both single and married adults. In addition to leisure-time physical activity the use of active transportation should also be promoted. These two types of physical activity are likely to be key to the attainment of appropriate levels of total physical activity of working-age people in the future. Taking steps to get working-age individuals physically active may not only enhance their health, but also have important implications for their own quality of life (health-, family-, or work-related) as well as the quality of life of their families and society as a whole.

## Supplemental Information

10.7717/peerj.12466/supp-1 Supplemental Information 1 Raw data.

10.7717/peerj.12466/supp-2 Supplemental Information 2 Questionnaire on the socio-economic status of people of working age.

10.7717/peerj.12466/supp-3 Supplemental Information 3 International Physical Activity Questionnaire (in English).

10.7717/peerj.12466/supp-4 Supplemental Information 4 Questionnaire on the socio-economic status of people of working age (in Polish).

10.7717/peerj.12466/supp-5 Supplemental Information 5 International Physical Activity Questionnaire (in Polish).

## Additional Information and Declarations

### Data Availability

The following information was supplied regarding data availability: The raw measurements are available in the Supplemental File.
