# Supplementary material for: Physical activity and socio-economic status of single and married urban adults: a cross-sectional study
Source: PeerJ. 2021 Nov 9;9:e12466. doi: 10.7717/peerj.12466 (PMC8588853; doi:10.7717/peerj.12466)
Supplement: Supplemental Information 2 [file peerj-09-12466-s002.docx]

**Questionnaire on the socio-economic status of people of working age**

Dear Sir or Madam! The purpose of this survey is to assess the socio-economic status of people of working age. The obtained results will be subject to statistical analysis and in the form of summary statements will be used only for scientific purposes. Thank you very much for participating in the research and your time.

1. Age:……………………………………………………………………………………………………………
2. Height [cm]:………………………………………………………………………………………………….
3. Weight [kg]:………………………………………………………………………………………………….
4. Sex:

❑ Woman

❑ Man

1. Education:

❑ Primary

❑ Secondary

❑ Higher

1. Job:

❑ Manual worker

❑ White collar worker

❑ Self-employed

❑ Student

❑ Unemployed

❑ Other (what?) ………………………………………………………………………………………………

1. Marital status:

❑ Single, widowed, divorced, separated, and legally separated persons as well as persons not living in a consensual union with another person, legally married persons but not forming a de facto marriage, and persons not living in a consensual union with another person

❑ Married persons and persons living with partners

1. Steady income:

❑ No

❑ Yes

1. Per capita income:

❑ Up to 130 USD

❑ 131-260 USD

❑ 261-390 USD

❑ 391-520 USD

❑ Over 520 USD

1. Disposable (net) income:

❑ No income

❑ Up to 52 USD

❑ 53-104 USD

❑ 105-156 USD

❑ Over 156 USD

1. Savings:

❑ Yes

❑ No

1. Indebtedness:

❑ Yes

❑ No
