# Supplementary material for: Physical activity and socio-economic status of single and married urban adults: a cross-sectional study
Source: PeerJ. 2021 Nov 9;9:e12466. doi: 10.7717/peerj.12466 (PMC8588853; doi:10.7717/peerj.12466)
Supplement: Supplemental Information 3 [file peerj-09-12466-s003.doc]

**INTERNATIONAL PHYSICAL ACTIVITY QUESTIONNAIRE**

**(August 2002)**

**SHORT LAST 7 DAYS SELF-ADMINISTERED FORMAT**

# **FOR USE WITH YOUNG AND MIDDLE-AGED ADULTS (15-69 years)**

The International Physical Activity Questionnaires (IPAQ) comprises a set of 4 questionnaires. Long (5 activity domains asked independently) and short (4 generic items) versions for use by either telephone or self-administered methods are available. The purpose of the questionnaires is to provide common instruments that can be used to obtain internationally comparable data on health–related physical activity.

***Background on IPAQ***

The development of an international measure for physical activity commenced in Geneva in 1998 and was followed by extensive reliability and validity testing undertaken across 12 countries (14 sites) during 2000. The final results suggest that these measures have acceptable measurement properties for use in many settings and in different languages, and are suitable for national population-based prevalence studies of participation in physical activity.

## *Using IPAQ*

Use of the IPAQ instruments for monitoring and research purposes is encouraged. It is recommended that no changes be made to the order or wording of the questions as this will affect the psychometric properties of the instruments.

## *Translation from English and Cultural Adaptation*

Translation from English is supported to facilitate worldwide use of IPAQ. Information on the availability of IPAQ in different languages can be obtained at [www.ipaq.ki.se](http://www.ipaq.ki.se/). If a new translation is undertaken we highly recommend using the prescribed back translation methods available on the IPAQ website. If possible please consider making your translated version of IPAQ available to others by contributing it to the IPAQ website. Further details on translation and cultural adaptation can be downloaded from the website.

# ***Further Developments of IPAQ***

International collaboration on IPAQ is on-going and an ***International Physical Activity Prevalence Study*** is in progress. For further information see the IPAQ website.

## *More Information*

More detailed information on the IPAQ process and the research methods used in the development of IPAQ instruments is available at [www.ipaq.ki.se](http://www.ipaq.ki.se/) and Booth, M.L. (2000). *Assessment of Physical Activity: An International Perspective*. Research Quarterly for Exercise and Sport, 71 (2): s114-20. Other scientific publications and presentations on the use of IPAQ are summarized on the website.

### INTERNATIONAL PHYSICAL ACTIVITY QUESTIONNAIRE

We are interested in finding out about the kinds of physical activities that people do as part of their everyday lives. The questions will ask you about the time you spent being physically active in the **last 7 days**. Please answer each question even if you do not consider yourself to be an active person. Please think about the activities you do at work, as part of your house and yard work, to get from place to place, and in your spare time for recreation, exercise or sport.

Think about all the **vigorous** activities that you did in the **last 7 days**. **Vigorous** physical activities refer to activities that take hard physical effort and make you breathe much harder than normal. Think *only* about those physical activities that you did for at least 10 minutes at a time.

1. During the **last 7 days**, on how many days did you do **vigorous** physical activities like heavy lifting, digging, aerobics, or fast bicycling?

_____ **days per week**

No vigorous physical activities ***Skip to question 3***

1. How much time did you usually spend doing **vigorous** physical activities on one of those days?

_____ **hours per day**

_____ **minutes per day**

Don’t know/Not sure

Think about all the **moderate** activities that you did in the **last 7 days**. **Moderate** activities refer to activities that take moderate physical effort and make you breathe somewhat harder than normal. Think only about those physical activities that you did for at least 10 minutes at a time.

1. During the **last 7 days**, on how many days did you do **moderate** physical activities like carrying light loads, bicycling at a regular pace, or doubles tennis? Do not include walking.

_____ **days per week**

No moderate physical activities ***Skip to question 5***

1. How much time did you usually spend doing **moderate** physical activities on one of those days?

_____ **hours per day**

_____ **minutes per day**

Don’t know/Not sure

Think about the time you spent **walking** in the **last 7 days**. This includes at work and at home, walking to travel from place to place, and any other walking that you have done solely for recreation, sport, exercise, or leisure.

5. During the **last 7 days**, on how many days did you **walk** for at least 10 minutes at a time?

_____ **days per week**

No walking ***Skip to question 7***

1. How much time did you usually spend **walking** on one of those days?

_____ **hours per day**

_____ **minutes per day**

Don’t know/Not sure

The last question is about the time you spent **sitting** on weekdays during the **last 7 days**. Include time spent at work, at home, while doing course work and during leisure time. This may include time spent sitting at a desk, visiting friends, reading, or sitting or lying down to watch television.

1. During the **last 7 days**, how much time did you spend **sitting** on a **week day**?

_____ **hours per day**

_____ **minutes per day**

Don’t know/Not sure

**This is the end of the questionnaire, thank you for participating.**
