# Supplementary material for: Physical activity and socio-economic status of single and married urban adults: a cross-sectional study
Source: PeerJ. 2021 Nov 9;9:e12466. doi: 10.7717/peerj.12466 (PMC8588853; doi:10.7717/peerj.12466)
Supplement: Supplemental Information 4 [file peerj-09-12466-s004.pdf]

Kwestionariusz statusu społeczno-ekonomicznego osób w wieku produkcyjnym

Szanowni Państwo, celem badania jest identyfikacja statusu społeczno-ekonomicznego osób w wieku produkcyjnym. Uzyskane wyniki zostaną poddane analizie statystycznej oraz w postaci zestawień zbiorczych posłużą wyłącznie celom naukowym. Bardzo dziękujemy za udział w badaniu i poświęcony czas.

1. Wiek:……………………………………………………………………………………………………
2. Wysokoś ciała [cm]:……………………………………………………………………………………
3. Masa ciała [kg]:…………………………………………………………………………………………
4. Płeć:
   - ❑ Kobieta
   - ❑ Mężczyzna
5. Wykształcenie:
   - ❑ Podstawowe i zasadnicze zawodowe
   - ❑ Średnie
   - ❑ Wyższe
6. Status zawodowy:
   - ❑ Pracownik fizyczny
   - ❑ Pracownik Umysłowy
   - ❑ Przedsiębiorca
   - ❑ Student
   - ❑ Bezrobotny
   - ❑ Inny (jaki?) ………………………………………………………………………………………
7. Stan cywilny:
   - ❑ Panna i kawaler, wdowa i wdowiec, rozwiedziony i rozwiedziona, separowany i separowana, osoby pozostające w separacji prawnej i nie tworzące związku kohabitacyjnego z inną osobą oraz osoby pozostające w prawnym małżeństwie, lecz nie tworzące już wspólnoty małżeńskiej, a także nie będące w związku kohabitacyjnym z inną osobą
   - ❑ Żonaty i zamężna oraz partner i partnerka
8. Stały dochód:
   - ❑ Nie
   - ❑ Tak
9. Dochód brutto na osobę na miesiąc:
   - ❑ Do 130 USD
   - ❑ 131-260 USD
   - ❑ 261-390 USD
   - ❑ 391-520 USD
   - ❑ Powyżej 520 USD
10. Dochód netto do dyspozycji na osobę:
    - ❑ Brak
    - ❑ Do 52 USD
    - ❑ 53-104 USD
    - ❑ 105-156 USD
    - ❑ Powyżej 156 USD
11. Oszczędności:
    - ❑ Tak
    - ❑ Nie
12. Zadłużenie:
    - ❑ Tak
    - ❑ Nie
