# Supplementary material for: Physical activity and socio-economic status of single and married urban adults: a cross-sectional study
Source: PeerJ. 2021 Nov 9;9:e12466. doi: 10.7717/peerj.12466 (PMC8588853; doi:10.7717/peerj.12466)
Supplement: Supplemental Information 5 [file peerj-09-12466-s005.pdf]

MIĘDZYNARODOWY KWESTIONARIUSZ AKTYWNOŚCI FIZYCZNEJ

Chciał(a)bym zadać kilka pytań dotyczących czasu spędzanego na czynnościach wymagających aktywności fizycznej. Dotyczą one wszystkich rodzajów aktywności fizycznej związane z życiem codziennym, z pracą i z wypoczynkiem. Pytania te zadajemy wszystkim, niezależnie od tego, czy ktoś uważa się za osobę aktywną fizycznie, czy też nie.

Pytania będą dotyczyć czynności związanych z aktywnością fizyczną w ciągu ostatnich 7 dni, tzn. od .... **(podać dzień tygodnia) **do wczoraj.

Proszę teraz pomyśleć o wszystkich czynnościach wykonywanych w ciągu ostatnich 7 dni w domu i w jego otoczeniu, w pracy zawodowej, związanych z przemieszczaniem się z miejsca na miejsce, np. drodze do pracy i z pracy, robieniu zakupów. Proszę także uwzględnić czynności wykonywane w czasie wolnym, tj. spacery, rekreacja, praca na działce, ćwiczenia fizyczne oraz sport. Najpierw zapytam Pana/ą o czynności wymagające dużego wysiłku fizycznego, następnie o czynności wymagające umiarkowanego, średniego wysiłku, a na koniec o spacery i inne czynności związane z chodzeniem oraz siedzeniem.

Na początek proszę przypomnieć sobie wszystkie czynności wymagające **intensywnego wysiłku fizycznego**, *wykonywane w ciągu ostatnich 7 dni.*

**Intensywny wysiłek fizyczny** wywołuje bardzo szybkie oddychanie i bardzo szybkie bicie serca

Intensywnego wysiłku fizycznego wymaga np. dźwiganie ciężkich przedmiotów, kopanie ziemi, aerobik, szybki bieg, szybka jazda rowerem. Interesują nas tylko czynności, które trwały **co najmniej 10 min. bez przerwy.**

1. Czy w ciągu **ostatnich 7 dni** wykonywał/a Pan/i czynności wymagające **intensywnego wysiłku fizycznego**?

   Tak – przez ile dni w ciągu ostatniego tygodnia? .... dni
   Nie → ***przejść do pytania pyt. 3***
   Nie wiem/Nie jestem pewien(a) → ***przejść do pytania pyt. 3***

2. Przeciętnie ile czasu wykonywał/a Pan/i czynności wymagające **intensywnego wysiłku fizycznego** w ciągu takiego dnia?

   .... minut dziennie
   Nie wiem/Nie jestem pewien(a)

A teraz proszę przypomnieć sobie wszystkie czynności wymagające **umiarkowanego (średniego) wysiłku fizycznego** *wykonywane w ciągu ostatnich 7 dni.,*

**Umiarkowany wysiłek fizyczny** prowadzi do trochę szybszego oddychania i trochę szybszego bicia

Umiarkowanego wysiłku fizycznego wymaga np. noszenie lżejszych ciężarów, jazda rowerem w normalnym tempie, gra w siatkówkę lub bardzo szybki marsz. Proszę jednak nie brać pod uwagę chodzenia. Chodzi znowu tylko czynności, które trwały **co najmniej 10 minut bez przerwy.**

3. Czy w ciągu **ostatnich 7 dni** wykonywał/a Pan/i czynności wymagające umiarkowanego, średniego wysiłku fizycznego?

Tak – przez ile dni w ciągu ostatniego tygodnia? .... dni

Nie → ***przejść do pytania pyt. 5***

Nie wiem/Nie jestem pewien(a) → ***przejść do pytania pyt. 5***

4. Przeciętnie ile czasu wykonywał/a Pan/i czynności wymagające **umiarkowanego wysiłku fizycznego** w ciągu takiego dnia?

.... minut dziennie

Nie wiem/Nie jestem pewien(a)

Teraz proszę przypomnieć sobie, ile czasu zajęło Panu/i **chodzenie** w ciągu **ostatnich 7 dni.** Interesuje nas chodzenie związane z pracą, chodzenie ulicą, np. po zakupy, do pracy, a także o spacery.
Chodzi znowu o chodzenie, które trwało **co najmniej 10 minut bez przerwy.**

5. Czy w ciągu **ostatnich 7 dni** chodził/a Pan/i **co najmniej 10 min. bez przerwy**?

Tak – przez ile dni w ciągu ostatniego tygodnia? .... dni

Nie → ***przejść do pytania pyt. 7***

Nie wiem/Nie jestem pewien(a) → ***przejść do pytania pyt. 7***

6. Przeciętnie ile czasu poświęcał/a Pan/i na **chodzenie lub spacery** w ciągu takiego dnia?

.... minut dziennie

Nie wiem/Nie jestem pewien(a)

A ile czasu w ostatnim tygodniu spędzał Pan/i **siedząc**? Tym razem **proszę uwzględnić tylko dni powszednie,** tzn. proszę pominąć sobotę i niedzielę. Chodzi np. o siedzenie przy biurku, siedzenie podczas odwiedzin u znajomych, podczas czytania, a także siedzenie lub leżenie podczas oglądania telewizji. Proszę uwzględnić czas spędzony na siedzeniu w domu, w pracy, w szkole, w pojazdach i w innych miejscach.

7. Biorąc pod uwagę **dni powszednie w ciągu ostatniego tygodnia**, ile zazwyczaj czasu w ciągu dnia spędzał/a Pan/i **siedząc**?

.... minut dziennie

Nie wiem/Nie jestem pewien(a)
